# Feline panleukopaenia virus in captive non-domestic felids in South Africa

**DOI:** 10.4102/ojvr.v83i1.1099

**Published:** 2016-06-09

**Authors:** Emily P. Lane, Helene Brettschneider, Peter Caldwell, Almero Oosthuizen, Desiré L. Dalton, Liza du Plessis, Johan Steyl, Antoinette Kotze

**Affiliations:** 1 Department of Research and Scientific Services, National Zoological Gardens of South Africa, South Africa; 2 Old Chapel Veterinary Clinic, Pretoria, South Africa; 3 Department of Genetics, University of the Free State, South Africa; 4 IDEXX Laboratories (Pty) Ltd., Onderstepoort, South Africa; 5 Department of Paraclinical Science, University of Pretoria, South Africa

## Abstract

An outbreak of feline panleukopaenia virus (FPLV) infection was diagnosed by pathology, electron microscopy and polymerase chain reaction (PCR) in vaccinated captive-bred subadult cheetahs in South Africa. Subsequent to this disease outbreak, 12 cases of FPLV diagnosed on histology were confirmed by PCR in captive African black-footed cat, caracal, cheetah, lion, ocelot and serval. Phylogenetic analyses of the viral capsid protein gene on PCR-positive samples, vaccine and National Center for Biotechnology Information (NCBI) reference strains identified a previously unknown strain of FPLV, present since at least 2006, that differs from both the inactivated and the modified live vaccine strains. A previously described South African strain from domestic cats and cheetahs was identified in a serval. Surveys of FPLV strains in South African felids are needed to determine the geographical and host species distribution of this virus. Since non-domestic species may be reservoirs of parvoviruses, and since these viruses readily change host specificity, the risks of FPLV transmission between captive-bred and free-ranging carnivores and domestic cats and dogs warrant further research.

## Introduction

Feline panleukopaenia virus (FPLV) infection, the first viral disease identified in cats, is one of the most common viral diseases of domestic cats (*Felis domesticus*). FPLVs are classified under the family Parvoviridae and are related to mink enteritis virus (MEV) and canine parvovirus (CPV) (Nakamura *et al*. 2001; Steinel *et al*. 2000; Truyen *et al*. 1996). It is highly contagious, affects both domestic and non-domestic felids and is transmitted by direct and indirect contact with infected animals and their secretions, especially faeces. Despite aggressive treatment it can be fatal in less than 24 hours. Like all parvoviruses, FPLV is extremely resistant and can survive for longer than one year in suitable environments (Nakamura *et al*. 2001).

FPLV-like viruses show a global distribution, infecting a wide range of felids including the cheetah (*Acinonyx jubatus*) (Van Vuuren *et al*. 2000), European wild cat (*Felis sylvestris sylvestris*) (Wasieri *et al*. 2009), lion (*Panthera leo*) (Chandran *et al*. 1993), African wild cat (*Felis lybica*), ocelot (*Leopardus pardalis*), puma (*Puma concolor*), Siberian tiger (*Panthera tigris altaica*), and spotted cat (*Leopardus tigrinus*) (Filoni *et al*. 2006). In addition, the virus has been reported in the mink (*Neovison vison*), arctic fox (*Vulpes lagopus*) and raccoon (*Procyon lotor*), and is genetically very similar but distinct from CPV-like viruses from dogs (*Canis familiaris*) and raccoons (Filoni *et al*. 2006; Parrish 1995; Truyen *et al*. 1996). CPV is an ancient canid pathogen that re-emerged as a domestic dog pandemic in the late 1970s in Europe, as a variant of FPLV or a closely-related parvovirus in non-domestic carnivores, most likely through a viral mutation that facilitated previously blocked entry to cells through the transferrin receptor type 1 (Allison *et al*. 2013; Belyi, Levine & Skalka 2010; Nakamura *et al*. 2001; Steinel *et al*. 2001). Although the original strain (CPV type 2) did not infect felids, CPV2s subsequently mutated into additional strains (CPV types 2a, 2b and 2c) that are reported to induce disease in cats (Hoelzer *et al*. 2008; Parrish *et al*. 1991; Steinel *et al*. 2000). Parvoviruses in non-domestic carnivores may represent a reservoir that periodically spills over into domestic carnivore populations or provide alternate evolutionary pathways that facilitate pandemics (Allison *et al*. 2012, 2013, 2014; Belyi *et al*. 2010). Both CPV and FPLV typically infect multiple hosts and passage through different hosts may favour selected viral capsid mutations that facilitate changed host specificity (Allison *et al*. 2014).

Molecular confirmation of FPLV in a diseased domestic cat and two diseased captive born cheetahs in 2000 challenged the perception that FPLV had disappeared from domestic cats in South Africa due to the increased incidence of CPV strains type 2a and b infecting cats in the 1980–1990s (Van Vuuren *et al*. 2000). While parvoviral infections have in the past been diagnosed clinically, by electron microscopy and histopathological examination in domestic and non-domestic felids, the advent of molecular diagnostic techniques provides a new opportunity to evaluate the genetic variability of FPLV in affected animals. This article reports the clinical features and pathology of an outbreak of FPLV in vaccinated captive cheetahs, as well as preliminary findings on the molecular epidemiology of FPLV in captive non-domestic felids in South Africa.

## Materials and methods

Sample collection date, type and locality, host species, age, sex and vaccination status as well as pathological disease diagnosis and molecular analysis results are displayed in Table 1. In October and November 2012, eleven 6–8-month-old captive-bred cheetah cubs from one cheetah breeding institution were presented to the Old Chapel Veterinary Clinic with acute inappetance, lethargy and vomiting. Five cubs died and were submitted to the National Zoological Gardens of South Africa (NZG) for necropsy examination. Tissue samples collected during necropsy examinations were preserved in 10% buffered formalin for histopathology, processed routinely and stained with haematoxylin-eosin. DNA extraction and molecular characterisation were performed on fresh intestine from these cubs (Cases 1–5).

**TABLE 1:** Sample collection date, type and locality; species, sex, age and vaccination history; diagnosis and molecular analysis of samples tested for of feline panleukopaenia virus (FPLV).

Case	Date	Intestine sample	Locality	Species	Sex	Age	Vaccination	Diagnosis	Molecular analysis
1	2012/10/25	F/F	NW	Cheetah	F	8 months	Killed and live vaccine	suspected FPLV	FPLV Clade 1
2	2012/11/01	F/F	NW	Cheetah	F	6 months	n/r	FPLV	FPLV Clade 1
3	2012/11/07	F/F	NW	Cheetah	M	6 months	Killed and live vaccine	FPLV	FPLV Clade 1
4	2012/11/12	F/F	NW	Cheetah	M	5 months	Killed and live vaccine	FPLV	FPLV Clade 1
5	2013/02/16	F/F	NW	Cheetah	M	9 months	Killed and live vaccine	FPLV	FPLV Clade 1
6	2013/03/14	F/F	L	Serval	M	3 months	Killed vaccine	Salmonellosis	FPLV Clade 1
7	2013/05/07	F/F	G	Cheetah	F	8 months	n/r	FPLV	FPLV Clade 1
8	2013/05/07	F/F	G	Cheetah	M	8 months	n/r	FPLV	FPLV Clade 1
9	2013/05/21	FFPE	NW	Cheetah	n/r	stillborn	n/r	suspected FPLV	Negative
10	2013/06/20	FFPE	FS	Serval	n/r	n/r	n/r	suspected FPLV	FPLV Clade 2
11	2013/09/20	F/F	n/r	Lion	n/r	n/r	n/r	suspected FPLV	Negative
12	2014/02/28	FFPE	NW	Ocelot	n/r	n/r	n/r	suspected FPLV	FPLV Clade 1
13	2014/04/17	FFPE	G	Lion	M	2 weeks	Not vaccinated	FPLV	Negative
14	2014/04/17	FFPE	NW	Lion	F	3 months	Not vaccinated	FPLV	FPLV Clade 1
15	2014/04/17	FFPE	G	Caracal	F	2 months	Not vaccinated	suspected FPLV	FPLV Clade 1
16	2014/04/29	F/F	n/r	Cheetah	M	6 months	Not vaccinated	FPLV	FPLV Clade 1
17	2014/05/02	Rectal swab	EC	Cheetah	M	4 months	n/r	FPLV	FPLV Clade 1
18	2014/05/09	F/F	NW	Cheetah	F	8 months	Not vaccinated	FPLV	FPLV Clade 1
19	2014/05/09	F/F	NW	Cheetah	F	8 months	Not vaccinated	FPLV	FPLV Clade 1
20	2014/05/23	FFPE	EC	Caracal	n/r	n/r	n/r	suspected FPLV	Negative
A	2006/06/28	FFPE	NW	Lion	M	1 month	Not vaccinated	FPLV	FPLV Clade 1
B	2007/07/10	F/F	NW	African black-footed cat	F	8 months	Not vaccinated	FPLV	FPLV Clade 1
C	2010/03/04	F/F	G	Cheetah	M	1 month	n/r	FPLV	Negative
D	2010/05/29	F/F	n/r	Lion	n/r	4.5 months	n/r	suspected FPLV	Negative
E	2011/01/24	F/F	G	Domestic Cat	F	2.5 months	Not vaccinated	suspected FPLV	Negative
F	2011/03/14	F/F	NW	Cheetah	M	12.5 years	n/r	suspected FPLV	Negative
i	2014/01/23	F/F	L	Puma	M	juvenile	n/r	trauma	Negative
ii	2014/02/28	F/F	NW	Leopard	M	10 months	n/r	Bloat	Negative
iii	2014/04/29	F/F	G	Ocelot	F	1.5 months	n/r	Diaphragmatic hernia	Negative

FFPE, formalin-fixed paraffin-embedded; F/F, fresh or frozen; EC, Eastern Cape; FS, Free State; G, Gauteng; L, Limpopo; NW, North West; WC, Western Cape; n/r, not recorded; M, male; F, female; FPLV, feline panleukopaenia virus.

Subsequent to the outbreak in cheetahs, animal carcasses, a rectal swab, formalin-fixed tissues or formalin-fixed paraffin-embedded tissues (FFPE) from various single or multiple cases in captive felids were submitted to the NZG for pathological examination and/or molecular diagnostic testing for FPLV infection by the owners, veterinarians and pathologists throughout South Africa (Cases 6–20). Submitted cases of suspected or confirmed FPLV based on clinical signs and typical macroscopic and histological findings, or diseases presenting with similar signs, included two caracals (*Caracal caracal*), seven cheetahs, three lions, one ocelot and two servals (*Leptailurus serval*). Clinical signs and pathological findings were not available from many of these cases. Before the 2012 cheetah outbreak molecular diagnostic confirmation of suspected FPLV cases was not readily available in South Africa. To investigate prior suspected or confirmed cases of FPLV molecular diagnostic testing was performed on archived tissue samples from felids in the NZG Biobank (Cases A–G), including both suspected and histologically typical FPLV cases. Cases were from Gauteng and North West provinces and included one African black-footed cat (*Felis nigripes*), a domestic cat, three cheetahs and two lions. Samples from a puma, a leopard (*Panthera pardus*) and an ocelot in which clinical and histological evidence of FPLV was absent (Cases i–iii) were included as negative control cases. DNA was also extracted from current Fellocell^®^ and Fel-O-Vax^®^ (Zoetis; Boehringer Ingelheim) vaccines. All the samples are archived at the NZG Biobank.

DNA from all the fresh, frozen samples and vaccines were extracted using the ZR Genomic DNA^TM^ Tissue MiniPrep kit (Zymo Research). DNA from FFPE samples were extracted using the MasterPure™ Complete DNA & RNA Purification Kit (Epicentre) following the manufacturers specifications. Molecular analysis of the parvovirus genome from all the isolates was conducted by amplification of a ~1300 base pair (bp) region of the viral capsid protein (VP2) gene (Wasieri *et al*. 2009). This large fragment was amplified in four polymerase chain reactions (PCRs), in order to reduce the fragment size for ease of amplification especially for FFPE samples. PCR cycling conditions were as follows: initial denaturation at 94 °C for 5 min, five cycles of denaturation at 94 °C for 30 s, annealing at 52 °C for 50 s and extension at 72 °C for 1 min, 10 cycles of denaturation at 94 °C for 30 s, annealing at 50 °C for 50 s and extension at 72 °C for 1 min and 15 cycles of denaturation at 94 °C for 30 s, annealing at 48 °C for 50 s and extension at 72 °C for 60 s. A final extension step of 72 °C for 5 min concluded the cycling. PCR was conducted in a DNA-free hood to ensure a lack of contamination, confirmed by the inclusion of a DNA-negative control (double distilled water) for each gene region.

All the positive amplicons, as determined by gel electrophoresis, were purified using Exonuclease I and Alkaline phosphatase (Thermofisher) treatment, cycle sequenced using the BigDye V3.1 (Applied Biosystems) chemistry and run on a 3130 genetic analyser (Applied Biosystems). The resulting sequences were analysed and aligned using the ClustalX (Thompson *et al*. 1997) function incorporated in MEGA5 (Tamura *et al*. 2011) and phylogenetically analysed using distance (Neighbor-Joining, NJ) in MEGA5 and Bayesian methods (BA) in MrBayes v3.1 (Huelsenbeck & Ronquist 2001). The best-fit model of sequence evolution was selected under the Akaike Information Criterion (AIC) in jModeltest (Posada 2008).

Phylogenetic analyses were performed on a 51-taxon VP2 gene dataset of 29 PCR-positive case samples, two vaccine strains and 30 National Center for Biotechnology Information (NCBI) parvovirus reference strains. Results from NJ (10 000 bootstrap replicates) and BA (1 million generations) are presented in Figure 4. The HKY+I model of sequence evolution was chosen as the best-fit model in jModeltest under the AIC. All the sequences generated during this study were submitted to the NCBI website under accession numbers (KP033232–KP033250).

## Results

**FIGURE 1:**
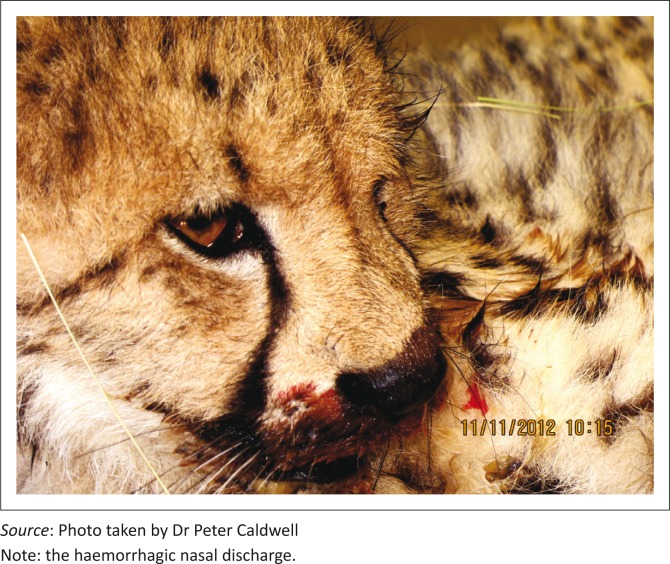
Feline panleukopaenia virus infection; cheetah, Case 8.

Clinical signs were described in detail only in the initial cheetah outbreak (Cases 1–5) and included pyrexia, haemorrhagic diathesis (Figure 1), hypersalivation, dehydration and inconsistent variably haemorrhagic diarrhoea. Some cubs showed polypnoea and marked aggression, thought to be due to pain. Five cubs died, despite aggressive treatment with intravenous and subcutaneous fluid replacement, anti-emetics, antibiotics, proton pump inhibitors, gastric cytoprotective agents, immune and appetite stimulants, multivitamins and a single dose of prednisolone (1%, 1 mg/kg). Oral electrolytes and small amounts of highly digestible cat food were syringe-fed to the cubs.

**FIGURE 2:**
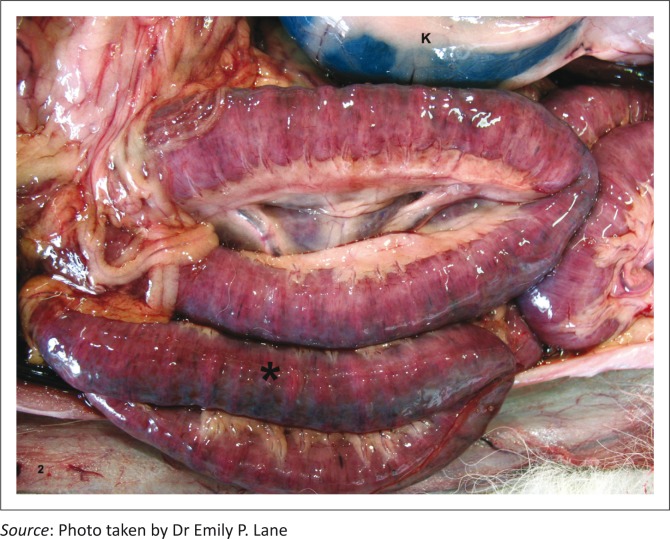
Feline panleukopaenia virus infection; abdomen, cheetah, Case 9. Severe intestinal serosal congestion and petechiation (*) of the intestinal loops adjacent to the kidney (K).

On necropsy examination, the cheetah cubs showed similar lesions including variable dehydration, mild acute diffuse tissue congestion, and moderate acute intestinal serosal congestion and haemorrhage (Figure 2). Variable numbers of petechiae and ecchymoses were present in other organs. Intestinal contents were scant, watery and bile stained. The intestinal mucosa was covered in a pale yellow-tan pseudomembrane.

**FIGURE 3:**
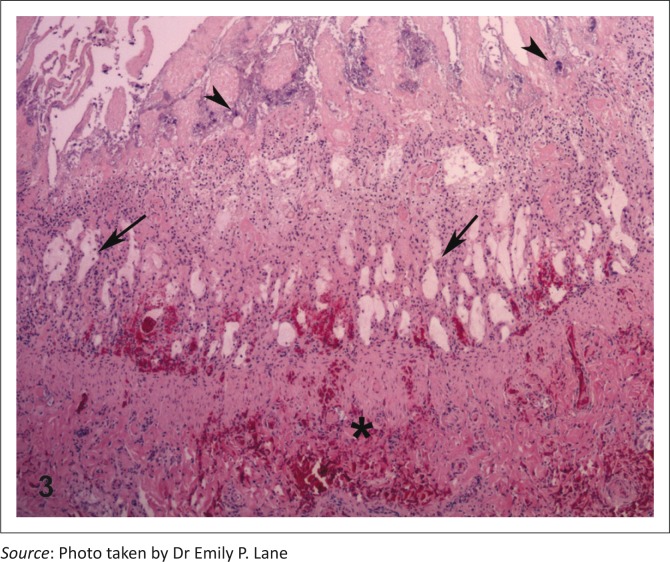
Feline panleukopaenia virus infection; intestine, cheetah, Case 9. Loss of intestinal crypt epithelial cells (arrows) is accompanied by mural haemorrhage (*) and bacterial colonies on the luminal surface (arrowheads). HE.

Histologically, extensive necrosis of the intestinal epithelium was accompanied by villous collapse, marked hyperplasia of scanty remaining epithelium, and a thick coat of fibrin, necrotic cellular debris and myriad bacteria on the mucosal surface (Figure 3). In addition, bone marrow pancytopenia and marked splenic, thymic, Peyer’s patch and lymph node lymphoid depletion were present. Colonies of bacteria of varied morphology occurred in the capillaries of many tissues associated with variable tissue necrosis. A heavy growth of *Escherichia coli* was obtained from the intestine from Case 1 and normal intestinal bacterial flora from Case 3. On negative contrast electron microscopy intestinal contents contained small numbers of parvovirus particles (Case 1).

The pathological lesions were consistent with FPLV infection with secondary bacterial enteritis and septicaemia. Additional pathological findings included moderate diffuse acute interstitial pneumonia with moderate multifocal acute pulmonary oedema (Cases 1, 3 & 4); multifocal acute moderate necrohemorrhagic bacterial gastritis as well as lingual and gastric candidiasis, mild serosanguinous hydropericardium and hydrothorax and acute moderate segmental renal tubular necrosis (Case 2); oesophageal and gastric candidiasis and acute mild multifocal pancreatic necrosis (Case 3); and mild acute multifocal necrotising aspiration pneumonia (Case 5). All the cubs were in good body condition at the time of death and showed mild to moderate diffuse adrenocortical hyperplasia.

**FIGURE 4:**
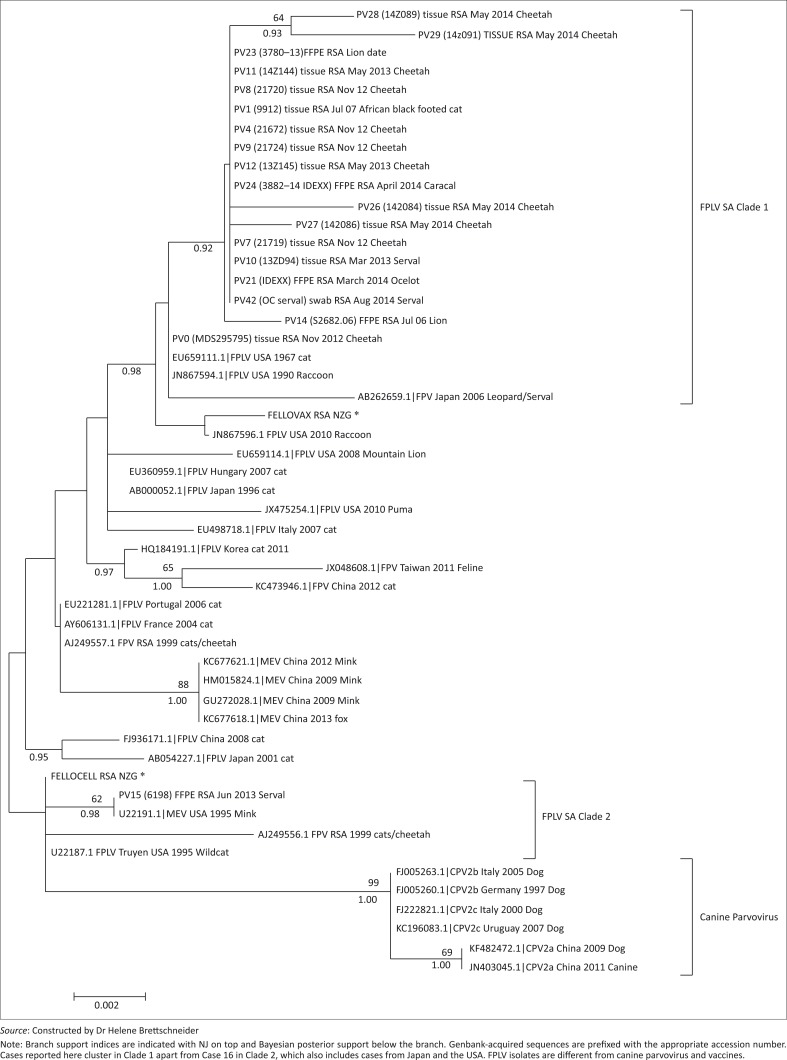
Feline panleukopaenia virus infection; neighbour-joining (NJ) tree of DNA extracted from South African felid samples, vaccine strains (*) and reference samples.

Phylogenetic analyses of the VP2 gene region of both vaccine strains and viruses from all cases were consistent with FPLV (99.99% similarity). All the samples from this study clustered separately from CPV (98% similarity) and clustered in two clades. Most of the positive cases (Clade 1) clustered closely (99.94% – 100%) with reference samples (EU659111.1, JN867594.1, AB262659.1) from carnivores in Japan and the United States of America (USA), and were similar to the Fell-O-Vax^®^ vaccine. Clade 2 is represented in the current dataset by one sample (Case 10), which clustered with previously described South African strains of FPLV isolated from a domestic cat (1999) and two cheetahs (2000) as well as an FPLV strain isolated from a wildcat and a mink in the USA (1995). This strain is 99.99% similar to the Fellocell^®^ vaccine (Figure 4).

## Discussion

Clinical features, pathology, electron microscopy and molecular diagnostic tests confirmed the presence of a previously unreported strain of FPLV in the cheetah cubs in the initial outbreak (Cases 1–4). This strain was also detected in archived samples from a lion that died in 2006 (Case A) and an African black-footed cat that died in 2007 (Case B), as well as in samples submitted from other captive felids that died throughout the country after November 2012, including cheetahs (Cases 7–9, 16–19), a caracal (Cases 15), a lion (Case 14), an ocelot (Case 12) and a serval (Case 6).

The testing of archived samples confirms that the strain from Clade 1 was present in felids in at least North West province before the initial cheetah outbreak and Clade 1 virus subsequently caused disease in unvaccinated felids in Gauteng and North West. Clade 1 strain apparently co-exists with the previously described strain (Van Vuuren *et al*. 2000) from Clade 2 at least in the serval (Case 10). Although Clade 1 clustered with samples from Japan and the USA, the absence of information on the distribution and phylogeny of FPLV in domestic and non-domestic felids in South Africa means that this should be interpreted with caution. The testing of more cases of FPLV in domestic and non-domestic felids (both captive and free-ranging) is needed to determine the geographical and host species distribution of the virus in South Africa and the relationship between these strains and those described from outside the country. This research would be greatly assisted by the provision of more detailed information on clinical features, histological findings and vaccination status of animals from which samples were submitted for pathological or molecular diagnostic samples. Cases were, as expected, equally prevalent in male and female animals, and were seen only in animals under eight months of age. Too few cases were tested to evaluate seasonal patterns of FPLV disease incidence.

Positive cases were found in animals from five of South Africa’s nine provinces; however, more extensive epidemiological surveys are needed to determine whether a geographical pattern is present, or if this was due to cheetah numbers differing between the provinces, or to clinicians and pathologists in these provinces submitting more samples than those from the other provinces. Should there be variation in the geographical distribution of the virus, this could inform provincial and national regulations regarding the movement of captive carnivores. Strict hygiene measures should in any case be implemented when animal attendants and visitors travel between national and international captive felid institutions. The survival of six of the 11 cheetah cubs in the initial outbreak was attributed to a lower infection load, intense monitoring and early aggressive treatment once the diagnosis had been made. Stress due to weaning and translocation may have been a predisposing factor in the variable susceptibility of these cubs to disease. However, FPLV can cause a spectrum of disease and the severity and clinical signs of the disease may be altered by the pathogenicity of variable secondary bacterial enteric infections (Parrish 1995).

Prior to the initial outbreak cases of FPLV were rarely experienced by the authors in captive carnivores in South Africa and molecular diagnostic techniques were not then readily available. The authors are unaware of any reports of the prevalence and strain type of FPLV in domestic cats or free-ranging felids in South Africa. Captive breeding of lion and other carnivores for hunting, ecotourism and conservation has grown significantly in the past decade (Lindsey *et al*. 2012). Intensive farming conditions, poor hygiene, a lack of vaccination and the ineffective exclusion of domestic carnivores from enclosures, surroundings and feeding stations may be increasing the incidence of FPLV in captive carnivores. The captive breeding of non-domestic carnivores may expose domestic dogs and cats to new variants of the virus, since non-domestic carnivores may be primary reservoirs for parvoviruses, the viruses are readily transmitted between species, and the viruses readily develop viral capsid protein mutations on passage through new hosts (Allison *et al*. 2012, 2013, 2014; Belyi *et al*. 2010). Further research is needed into the risks and patterns of FPLV transmission between domestic and both captive-bred and free-ranging non-domestic carnivores.

The source of the initial outbreak in the cheetahs remains uncertain. However, molecular characterisation of the strain indicated that the disease was not caused by CPV or inadequate inactivation of the inactivated vaccine, nor by unexpected pathogenicity of the live vaccine. Although vaccine viability was not tested, the failure of the live vaccine to induce immunity may have played a role in the outbreak. Vaccine failure may have been related to the loss of cold chain management during a 2012 transport strike. *Ante mortem* serological tests for FPLV antibodies were not available, so the immunity of the affected and non-affected animals could not be determined. Apart from the animals that were vaccinated during the transport strike in 2012, all other positive cases occurred in unvaccinated felids. The vaccination of domestic cats prevents disease due to FPLV as well as CPV 2b and 2c (Chalmers *et al*. 1999; Jack *et al*. 2014). Maternal immunity lasts between 2 and 4.5 months and domestic cats are therefore usually affected at 3–5 months of age. Inactivated (killed) vaccines can be used in pregnant queens, kittens under 4 weeks old and debilitated cats (Parrish 1995). Inactivated vaccines have also been proven to be safe and induce high persistent levels of serum antibodies to FPLV in cheetahs (Wack *et al*. 1993). Immunisation with modified live vaccines of adult domestic cats is thought to confer lifelong immunity; however, FPLV vaccines are not registered for use in other felids and their efficacy against different virus variants, particularly in non-domestic felids, is not well documented. A modified live vaccine induced a significant immune response in tigers but not lions (Risi *et al*. 2012). Despite this, vaccine regimens using a combination of inactivated (dams in late pregnancy and cubs at eight and 12 weeks of age) and live vaccines (cubs at 16 weeks of age) reduce cub mortality and have been proven to be largely safe and effective in captive cheetah populations (Munson & Citino 2005; P. Caldwell, personal communication 2015). Other cheetah cubs on the same property during and since the 2012 outbreak survived. The Clade 1 strain isolated from vaccinated animals was different from the modified live vaccine strain, so disease due to the vaccine strain was ruled out. Further research is needed to determine the relationship, if any, between disease in cheetahs, domestic cats and serval caused by Clade 2 viral strains.

Following the 2012 outbreak, control measures employed on the cheetah breeding facility were effective and no further deaths occurred. The enclosures were sprayed several times in the first week using high-pressure delivery systems for Parvoclean (Kyron), F10 products (Health and Hygiene [Pty] Ltd) or swimming pool chlorine (2.67 g/L water). Biosecurity measures included staff movement from clean to infected enclosures for feeding and inspections; new and frequently changed cleaning cloths as well as overalls and boots for all staff; hand gels and body sprays of F10 products used when moving between enclosures.

Positive cases were detected from fresh and frozen tissue, a faecal swab and FFPE intestinal samples from 16 animals clinically and pathologically diagnosed as suffering from FPLV or suspected FPLV (Table 1). Three cases without macroscopic and histological evidence of FPLV tested negative (Cases i–iii). However, eight animals (Cases 9, 11, 13, 20, C–F) with histological lesions suggestive of FPLV tested negative. Intestinal and lymphoid tissue histological lesions are considered pathognomonic for FPLV (Brown, Bake & Barker 2007), therefore these may represent false negative test results. Unfortunately, immunohistochemical tests for FPLV antigen were not available to verify the histological or PCR results. The amount of virus in the intestine may vary temporally, since viral antigen is rarely detectable by immunohistochemistry once clinical signs and intestinal epithelial necrosis are present (Brown, Bake & Barker 2007). Nonetheless, FPLV DNA was detected in many of these clinical cases where intestinal necrosis was present without intestinal viral inclusions. False negatives may also occur when the presence of virus is below the level of detection, when DNA is degraded by prolonged storage in either the freezer or in formalin (Srinivasan, Sedmak & Jewel 2002) or through the use of non-buffered formalin as a fixative (Gilbert *et al*. 2007). Nonetheless, archived FFPE tissue samples were found in this study to be a valuable test sample. As previously described (Errington, Jones & Sawyer 2014), tissue swabs may be an adequate sample for molecular analysis but since only one swab was tested this should be further evaluated with paired tissue samples. The discordance in the incompletely vaccinated serval diagnosed pathologically with salmonellosis with FPLV DNA in the intestine could have been caused by typical FPLV lesions being obscured by secondary opportunistic bacterial infections or the presence of FPLV infection without FPLV-induced disease (Parrish 1995). In addition, a false positive test result due to inadvertent contamination of the test sample with FPLV DNA is possible.

Although infections with viruses of the feline parvovirus subgroup have been reported before in several wild carnivore species, including African felids, this study reveals the first in-depth insight into strain diversity within captive felids in South Africa and the first documented cases of FPLV infection in the African black-footed cat and caracal. Further surveys are needed to determine the geographical and host species distribution as well as the genetic variability of FPLV in these species. Vaccine failure, inadequate vaccination and stress may be predisposing factors in the susceptibility to disease. Retention of the current vaccination programme as well as good hygiene practices at breeding and rearing facilities is recommended for the prevention and control of the disease.
